# Cross-sectional study comparing public and private hospitals in Catalonia: Is the practice of routine episiotomy changing?

**DOI:** 10.1186/s12913-015-0753-z

**Published:** 2015-03-11

**Authors:** Ramón Escuriet, María J Pueyo, Mercedes Perez-Botella, Xavi Espada, Isabel Salgado, Analía Gómez, Herminia Biescas, Isabel Espiga, Joanna White, Rosa Fernandez, Josep Fusté, Vicente Ortún

**Affiliations:** Directorate-General for Health Planning and Research, Ministry of Health of the Government of Catalonia, Barcelona, Spain; Department of Experimental and Health Sciences, Universitat Pompeu Fabra (UPF), Barcelona, Spain; Consorci Sanitari Integral, Hospital General de l’Hospitalet, L’Hospitalet de Llobregat, Barcelona, Spain; University of Central Lancashire, School of Health, Midwifery, Neonatal and Sexual Health Division, Preston, UK; Institut Català de la Salut, Unitat Atenció a la Salut Sexual i Reproductiva, Granollers, Spain; Fundació Hospital Asil de Granollers, Granollers, Spain; Women’s Health Observatory, Subdirectorate for Quality and Cohesion, Ministry of Health, Social Services and Equality, Madrid, Spain; Centre for Research in Anthropology (CRIA-IUL), Lisbon, Portugal; Visiting Fellow, King’s College, London, UK; Public Health Agency of Catalonia, Maternal and Infant Health Programme, Government of Catalonia, Barcelona, Spain; The Union, Health and Social Entities Association, Barcelona, Spain; Universitat Pompeu Fabra (UPF), Faculty of Economic and Business Sciences, Barcelona, Spain

**Keywords:** Episiotomy, Perineal trauma, Maternity services organisation

## Abstract

**Background:**

In Spain, the Strategy for Assistance in Normal Childbirth (SANC) promoted a model of care, which respects the physiological birth process and discards unnecessary routine interventions, such as episiotomies. We evaluated the rate of episiotomy use and perineal trauma as indicators of how selective introduction of the SANC initiative has impacted childbirth outcomes in hospitals of Catalonia.

**Methods:**

Cross-sectional study of all singleton vaginal term deliveries without instrument registered in the Minimum Basic Data Set (MBDS) of Catalonia in 2007, 2010 and 2012. Hospitals were divided into types according to funding (public or private), and four strata were differentiated according to volume of births attended.

Episiotomies and perineal injury were considered dependent variables. The relationship between qualitative variables was analysed using the chi-squared test, and Student’s *t*-test was used for quantitative variables. Comparison of proportions was performed on the two hospital groups between 2007 and 2012 using a Z-test. Logistic regression models were used to analyse the relationship between episiotomy or severe perineal damage and maternal age, volume of births and hospital type, obtaining odds ratios (OR) and 95% confidence intervals (CI).

**Results:**

The majority of normal singleton term deliveries were attended in public hospitals, where maternal age was lower than for women attended in private hospitals. Analysis revealed a statistically significant (*P* < 0.001) decreasing trend in episiotomy use in Catalonia for both hospital types. Private hospitals appeared to be associated with increased episiotomy rate in 2007 (OR = 1.099, CI: 1,057–1,142), 2010 (OR = 1.528, CI: 1,472–1,587) and 2012 (OR = 1.459, CI: 1,383–1,540), and a lower rate of severe perineal trauma in 2007 (OR = 0.164, CI: 0.095–0.283), 2010 (OR = 0.16, CI: 0.110–0.232) and 2012 (OR = 0.19, CI: 0.107–0.336). Regarding severe perineal injury, when independent variables were adjusted, maternal age ceased to have a significant correlation in 2012 (OR = 0.994, CI: 0.970–1.018).

**Conclusions:**

Episiotomy procedures during normal singleton vaginal term deliveries in Catalonia has decreased steadily since 2007. Study results show a stable incidence trend below 1% for severe perineal trauma over the study period.

## Background

Recent evidence has led to publication of recommendations about best practice in supporting normal childbirth. These recommendations focus on a model of care that centres on women’s needs, respects the physiological birth process and discards unnecessary routine interventions, such as episiotomies.

Episiotomy was originally incorporated as a routine clinical practice [1] to reduce the risk of foetal distress and perineal damage. The intervention is routinely recorded in childbirth procedure documentation, together with diagnosis of perineal trauma. Analysis of both phenomena is widely used to assess quality in maternity care [2-5].

After many years of the routine practice of episiotomy, the document “Appropriate Technology for Birth” published by the World Health Organisation (WHO) [6] acknowledged that routine use of the procedure was unjustified and recognised the importance of informing women about the procedure and respecting their wishes. In addition, systematic reviews [7,8] have concluded that routine use of episiotomy does not benefit the mother or infant. These developments have led to a change in the recommendations made by scientific bodies; a more selective use of the intervention is now favoured. Other studies have revealed the economic cost of episiotomy [9] and the economic impact of a reduction in its use [10,11].

Recommendations concerning the selective use of episiotomy published in 2006 by the American College of Obstetricians and Gynecologists (ACOG) [12] revealed that medial episiotomies were associated with a greater risk of third- and fourth-degree tears when compared with mediolateral episiotomies, and confirmed that routine episiotomy does not prevent perineal damage or associated incontinence. Other guidelines recommending restrictive use of episiotomy have emerged, such as those developed jointly in 2007 by the National Institute for Health and Care Excellence (NICE) and the Royal College of Obstetricians and Gynaecologists (RCOG) [13]. In Spain, a clinical practice guideline [14] was published in 2010, promoted by the Ministry of Health, Social Services and Equality (MHSSE) as part of its Strategy for Assistance in Normal Childbirth (SANC) within the Spanish National Health Service [15].

While clinical practice guidelines recommend practices based on the best available evidence, their everyday implementation is not without its challenges. Some health service providers have very strict protocols that are difficult to access and review, and some providers have adopted practices over the years that have become ingrained and are difficult to change. There are also economic drivers that leave little room for change [16-18]. The importance of integrating scientific evidence into clinical practice so as to improve care is well recognised; however, translation of knowledge into practice does not generally happen automatically. Strategies are often required to implement good clinical practice and adapt protocols to emerging knowledge [19].

Quality indicators, such as the rate of third- and fourth-degree perineal tears, are used to evaluate the quality of maternity services [20]. These are based on published clinical guidelines and are of paramount importance when comparing different models of care [21]. In Spain, introduction of the SANC in 2008 promoted changes in the model of care, orienting maternity care towards the needs of women and their families. Adoption of this strategy in Catalonia led to a project that provided funds to 32 hospitals to help them improve their labour and delivery rooms, to improve the information given to women so as to encourage them to participate in decision making about delivery, and to enhance staff training in the area of promoting natural childbirth. SANC recommendations have enhanced the incorporation of good clinical practice in normal deliveries, such as the selective use of episiotomy.

Several years after the introduction of SANC in Catalonia, it is important to review the initiative and understand its impact. For that purpose, an evaluation of the care provided during normal childbirth is underway. As part of this evaluation, we are analysing complied data about the structure and organisation of hospitals, obtained through a national database containing information about each hospital that provides maternity care. In addition, we are conducting visits to those hospitals that have received funds (accredited hospitals), and a series of indicators related to care processes and outcomes in relation to normal deliveries have been developed to assess performance. Analysis of data so far shows some differences between public and private hospitals in certain aspects of service organisation and outcomes [22]. Evolution of the rate of episiotomy use and perineal trauma in singleton vaginal term deliveries without instrumentation provides an important indication of how the selective use of episiotomy has impacted outcomes in both public and private hospitals.

The current study was therefore developed as part of the evaluation of care provided during normal births in Catalonia, with the following objectives:

- To determine trends of the global episiotomy rate in singleton vaginal term deliveries without instrument in Catalonia
- To identify trends of the global severe perineal trauma rate (third- and fourth-degree tears) in singleton vaginal term deliveries without instrument in Catalonia

The current recommended rate of episiotomies varies between less than 30% [10] and less than 15% [8]. As a standard reference in this work, we adhered to the WHO recommended episiotomy rate for comparison purposes, which is between 5% and 20%.

## Methods

We performed a cross-sectional study of all singleton vaginal births at term without instrumentation registered in the Minimum Basic Data Set (MBDS) of Catalonia in 2007, 2010 and 2012. These three time points were chosen to assess trends of the global rate of episiotomy and severe perineal trauma, from 1 year prior to the publication of SANC (2007) and for subsequent years (2010 and 2012), corresponding to 2 and 4 years after publication of the strategy, respectively.

The MBDS is mandatory for all public hospitals and provides the basis for state funding. Its use has been extended to private hospitals with a good record of diagnoses and procedures. The MBDS compiles information from 44 public and 20 (out of a total 27) private hospitals, thereby representing data for 98% of all births in Catalonia.

### Data collection

We first identified all singleton term deliveries (between 37 and 42 weeks’ gestation) registered in the MBDS during the three time points. Included births met the following criteria: vaginal delivery with no accompanying principal or secondary codes for C-section, forceps, vacuum or unspecified instrumental delivery. Included births were then attributed to the hospital where they were attended.

The unit of analysis in our study was the hospital, with the presumption that the nature of the health care organisation influences the probability that an obstetric intervention will be carried out. For our analysis, hospitals were divided into two types according to funding (public or private) and four strata were differentiated according to the volume of births attended (stratum 1 (S1): <600 births pa, stratum 2 (S2): 600–1200 births pa, stratum 3 (S3): 1201–2400 births pa, stratum (S4): >2400 births pa).

Episiotomies and severe perineal trauma were considered dependent variables and their analysis was restricted to singleton vaginal births at term without instrument. Episiotomies were recorded as yes/no and severe perineal trauma was analysed according to degree of perineal tearing, with third- and fourth degree tears being grouped together into a category of “severe perineal trauma” whether tearing occurred spontaneously or after an episiotomy had been performed.

Independent variables were maternal age, type of hospital funding and strata of attended births. A descriptive analysis for each group of hospitals and strata was carried out using the mean and confidence interval (95%) for each dependent variable.

To determine whether the proportion of episiotomies performed had changed since introduction of the SANC strategy, a comparison of proportions was performed on the two hospital types between 2007 and 2012 using the Z-test (level of significance α = 0.05).

The relationship between qualitative variables was analysed using the chi-squared test and Student’s *t*-test was used for quantitative variables. Finally, logistic regression models were used to analyse the relationship between episiotomy or severe perineal damage and maternal age, volume of births and type of hospital (private or public).

### Ethical approval

This study was exempt from review by the Ministry of Health, Government of Catalonia Ethics Committee because it used publicly available, anonymised data.

## Results

**Figure 1:**
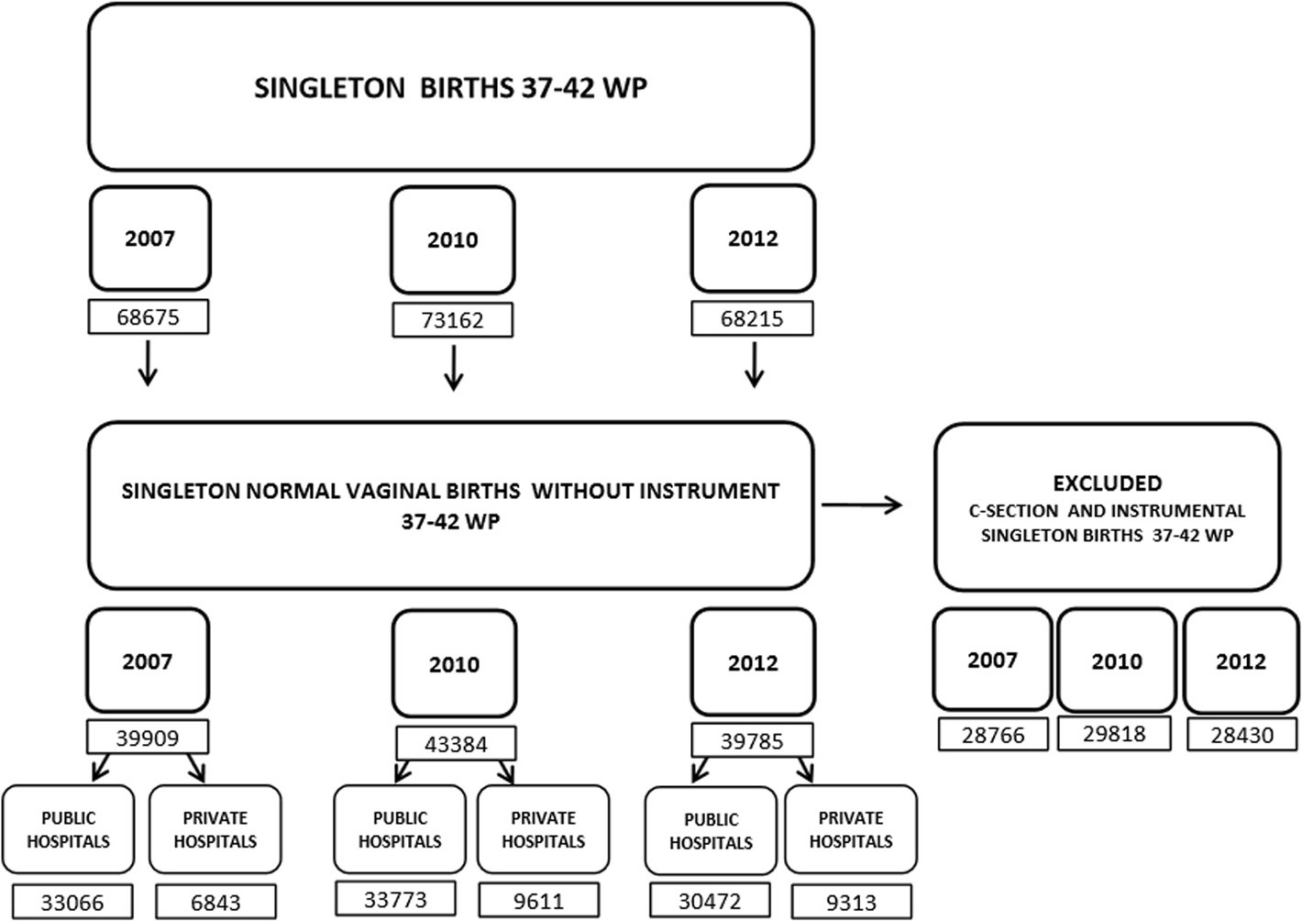
**Births distribution in two types of hospital.**

Singleton vaginal term deliveries without instrument represented more than half of all singleton term births, most of which were attended in public hospitals in Catalonia over the three time periods studied. Figure 1 shows the distribution of both included and excluded births.

Average maternal age was also calculated and analysed in relation to hospital type. This analysis revealed that the age of women experiencing singleton vaginal term deliveries without instrument was lower in public hospitals than that of women giving birth in private hospitals (Table 1).

**Table 1:** **Distribution and average maternal age in singleton vaginal births at term without instrument (37–42 wks)**

**YEAR**	**Public hospitals**		**Private hospitals**	
	**% of births attended**	**Average marternal age**	**% of births attended**	**Average maternal age**
**2007**	82.85%	29,73(SD 5,51)	(17.14%)	32,9 (SD 3,84)
**2010**	77.84%	30,08 (SD 5,57)	(22.15%)	33,32 (SD 3,82)
**2012**	76.59	30,45 (SD 5,63)	(23.40%)	33,65 (SD 3,88)

**Figure 2:**
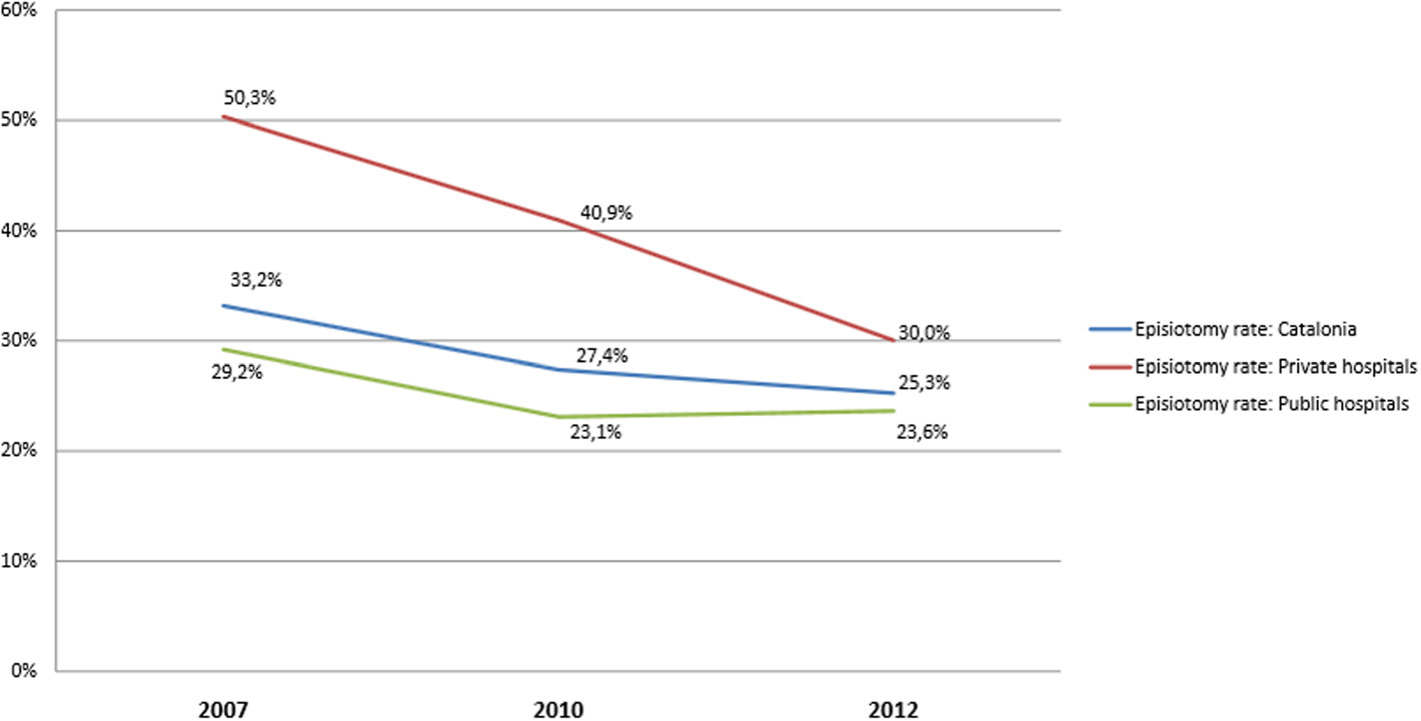
**Tendency of the evolution of the episiotomy rate.**

Figure 2 shows a downward trend in the global episiotomy rate in Catalonia and also in both hospital groups. All three indicators show a significant decrease *(P <* 0.001) since 2007. The most marked downward trend can be observed in private hospitals, which may be explained by the fact that their starting rates (prior to adoption of the new strategy) were markedly higher and these rates have significantly decreased over the last 5 years.

**Figure 3:**
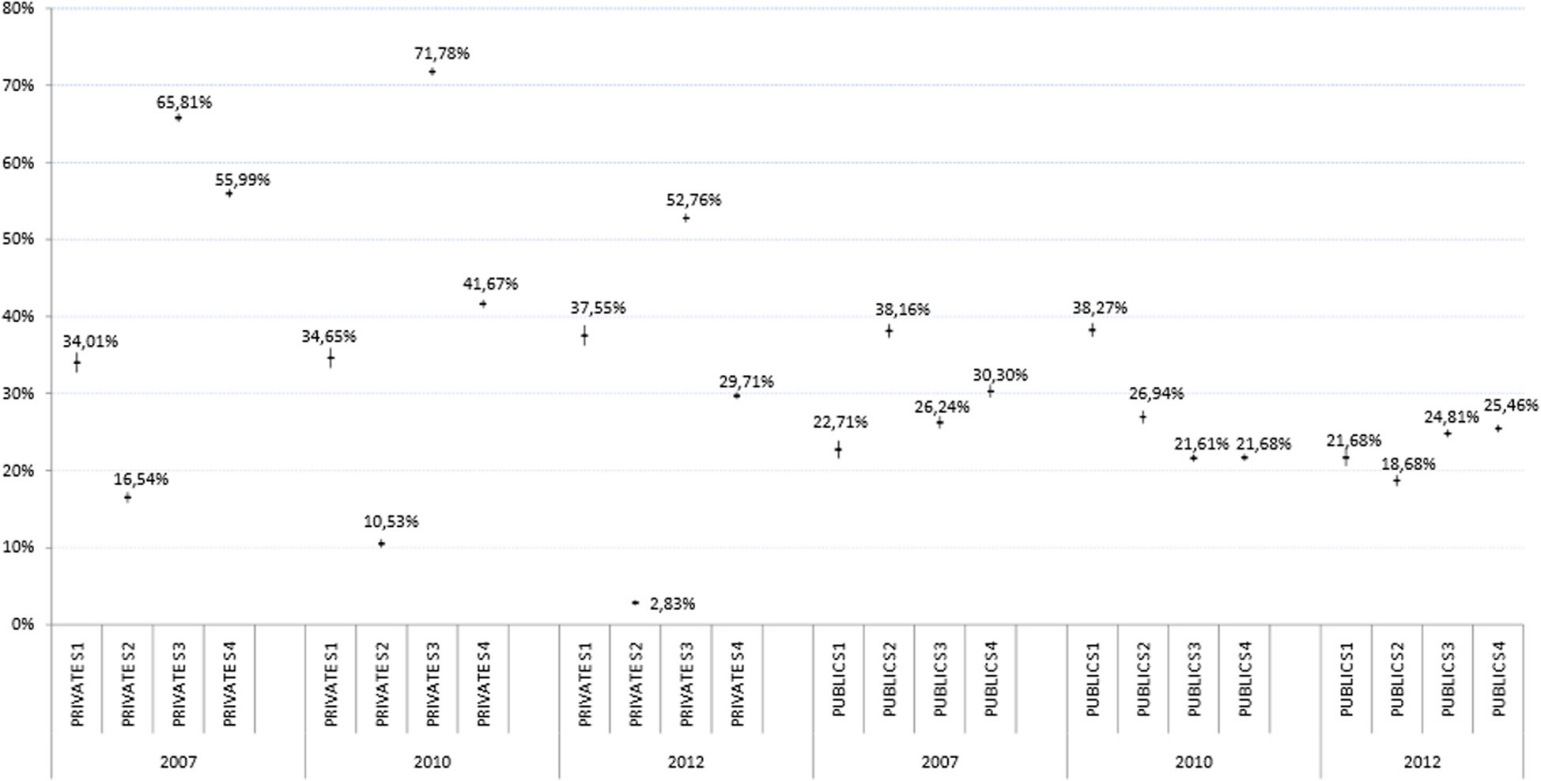
**Distribution of episiotomy rates by type of hospital and stratum in the tree times point.**

**Figure 4:**
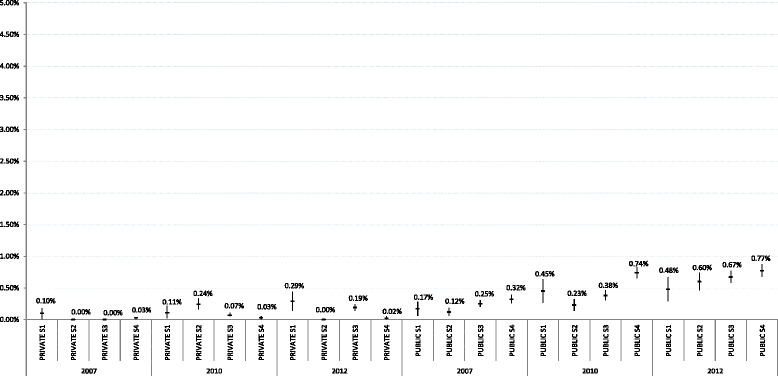
**Distribution of severe tear rates by type of hospital and stratum in the tree times point.**

The distribution of episiotomy rates and severe (third- and fourth-degree) perineal tears can be seen in Figures 3 and 4. Hospitals in both groups (private and public) were combined by strata according to their volume of births, and percentages and confidence intervals were calculated for episiotomies and severe perineal trauma for all births occurring in each stratum. Figure 3 shows a nonuniform distribution in the episiotomy rate, with a higher variability observed between strata in private hospitals; a more elevated rate is evident in S3 of this group across the three time points reviewed. It can be seen that severe perineal trauma remained below 1% in both groups and in all strata over the 3 years analysed.

Multivariate analysis (Table 2) shows a significant correlation between the use of episiotomy, private hospitals and the volume of births over the study period. Private hospitals were associated with an increased rate of episiotomy and a lower rate of severe perineal trauma. When independent variables are adjusted, maternal age ceases to have a significant correlation. Although both dependent variables were initially positively associated with the volume of births, this association did not reach significant levels in 2012.

**Table 2:** **Episiotomy and severe perineal tear association with maternal age, type of funding and volume of activity of the hospital**

		**Maternal age**		***P***	**type of hospital (public or private)**		***P***	**Volume activity**		***P***
		Odds R	(CI)		Odds R	(CI)		Odds R	(CI)	
2007	Episiotomy	0,977	0,973-0,980	*0,000*	1,099	1,057-1,142	*0,000*	1,139	1,118-1,161	*0,000*
	Perineal tears 3-4°	0,984	0,961-1,008	*0,189*	0,164	0,095-0,823	*0,000*	1,365	1,154-1,615	*0,000*
2010	Episiotomy	0,97	0,966-0,973	*0,000*	1,528	1,472-1,587	*0,000*	1,05	1,030-1,070	*0,000*
	Perineal tears 3-4°	0,991	0,975-1,008	*0,309*	0,16	0,110-0,232	*0,000*	1,171	1,039-1,320	*0,000*
2012	Episiotomy	0,971	0,967-0,976	*0,000*	1,459	1,383-1,540	*0,000*	1,136	1,105-1,168	*0,000*
	Perineal tears 3-4°	0,994	0,970-1,018	*0,628*	0,19	0,107-0,336	*0,000*	1,054	0,890-1,249	*0,540*

## Discussion

This study is part of an ongoing, wider evaluation of maternity care in Catalonia, in the context of the SANC strategy to promote natural childbirth and following the development of several initiatives to promote best clinical practice. The overall aim of the evaluation is to enhance knowledge about the use of obstetric interventions, the organisation of maternity care, and women’s satisfaction with the care received during pregnancy and delivery. For this purpose, visits have been made to the 32 hospitals that have adopted SANC, a survey of women’s experiences is being conducted, and a series of indicators about the use of obstetric interventions in normal singleton vaginal births are being assessed.

The data used in the current study were generated from an existing birth record database owing to its ease of access and consistency in data entry [23]. We investigated the use of episiotomy and occurrence of severe perineal damage, as defined above, as indicators of the quality and effectiveness of maternity care [2,3,5]. The rationale for this analysis was that studying trends in relation to these indicators over a period of time could provide some measure of the impact on clinical practice of implementing the SANC recommendations.

Throughout the period studied, maternal age as an independent variable nearly reached statistical significance in the prediction of severe perineal trauma; younger age appeared to be associated with a higher incidence risk. The type of hospital where the birth occurred was found to be associated with either increased use of episiotomy or increased incidence of perineal damage. Volume of births was an independent variable for the prediction of episiotomy from 2007 to 2012, at which point it ceased to predict severe perineal damage. This finding is consistent with another study where the likelihood of perineal laceration did not differ significantly by birth volume [24].

Using 2007 as a reference point, the results of this study confirm that the use of the episiotomy has decreased steadily in all Catalonian hospitals and that the SANC recommendations for selective use of this intervention are being followed. This finding strengthens other recommendations [25] regarding the importance of developing clinical guidelines and education programmes nationwide that target providers, encouraging them to change their practices of episiotomy use. This is especially important because restrictive use of this intervention does not affect neonatal outcomes [26] and is associated with reduced blood loss [27]. A good example of such strategies is the programme for midwives and obstetricians developed and implemented in Norway, which saw a reduction in the incidence of third- and fourth-degree tears [28]. On the other hand, a recent study on the performance of episiotomies in the United States questioned whether systematic reviews can change practice. The results of this study showed a gradual reduction in the episiotomy rate and concluded that it was negative obstetric outcomes associated with routine performance of the intervention that prompted professionals to change protocols and align them with recommendations arising from scientific evidence [29,30]. Similar conclusions have been reached in France [31], Finland [32] and Spain [33].

Existing evidence indicates that prophylactic nominal episiotomy does not prevent the risk of severe perineal damage [7]. In addition, the risk of subsequent severe perineal trauma for women who have previously undergone episiotomy is no higher than that for other women, and therefore episiotomy to prevent subsequent trauma is not justified [13-15]. The results of our study showed a stable incidence trend for severe perineal trauma over the period examined (below 1%). However, a marked difference between the two types of hospitals was observable. A study published in 2013 [34], which examined morbidity associated with the use of episiotomy, provided new information that complements existing knowledge generated from Carroli’s systematic review [7], thus reinforcing the recommendation to restrict episiotomy use and confirming that this approach reduces pain and dyspareunia without increasing urinary or faecal incontinence.

As part of the wider evaluation mentioned above, qualitative information was collected via semi-structured interviews with senior managerial staff in the 32 public hospitals that had obtained funding to implement SANC. In these interviews, some key aspects relating to the organisation of services were explored, in particular the organisation of maternity care. A common denominator in all hospitals was identification of the midwife as the lead carer for women throughout the childbearing continuum, including birth. This information is very valuable, permitting us to identify a factor in care delivery that is not recorded in official databases or hospital record systems and confirms that the majority of normal vaginal births are attended by midwives.

Finally, it is important to remember that recommendations for episiotomy use in Catalonia are framed within a strategy (SANC) that supports a model of care within which birth is considered a normal physiological process. The adoption of this model has entailed a series of changes, including physical improvement of the hospitals involved as well as pressure from local government encouraging providers to modify clinical practices that are not supported by scientific evidence.

Procedural changes within hospitals associated with SANC appear to have contributed toward modification of some obstetric practices, such as the routine use of cardiotocography during labour that allows women more freedom of movement during the second stage of labour, water use during the first and second stages of labour, progressive introduction of alternative therapies during labour, and alternative birth positions. All of these changes may have contributed to some degree to the steady fall of episiotomy rates in Catalonia. In light of these study results, introduction of indicators that measure these new developments in birth record databases is recommended, as a means of both documenting and promoting a model of maternity care that truly considers and evaluates birth as a normal physiological process [35].

We were unable to assess current rates of perineal trauma for singleton vaginal births at term when an episiotomy had been performed. It would be of interest to investigate the incidence of perineal trauma following episiotomy.

It would also be interesting to investigate other contributing factors and their relationship with the type of health care professional attending the birth, characteristics of the hospital where the birth took place, and maternal characteristics.

Existing data regarding diagnoses and obstetric interventions only refer to undesirable health outcomes. However, as already noted, it would be beneficial to record results about optimal health outcomes, such as intact perineum after normal vaginal delivery. This would permit a broader understanding of birth outcomes and shift the current focus on negative outcomes to a more positive examination of maternity care related to normal birth outcomes.

### Limitations

This study aimed to evaluate the impact of national guidelines and recommendations on clinical practice. For this purpose, the hospital was taken as the unit of analysis, obviating potential different interprofessional practices.

We are aware that the characteristics of women attending private or public hospitals may vary, which could potentially affect the results. Also, information about parity for every woman included in the study was not available.

Another important limitation lies in the nature of the available data, which does not permit a distinction between spontaneous severe perineal damage or that arising after an episiotomy has been performed.

Caution is essential in the interpretation of these data, as underreporting of severe perineal damage could skew the results [29]. Nevertheless, there is evidence that maintenance of records relating to hospital activities and accurate coding of information have significantly improved in the Catalonian region, especially in publicly funded hospitals.

## Conclusions

The rate of episiotomies performed during singleton vaginal term deliveries without instrument has decreased since 2007 in both private and public hospitals in Catalonia, and severe perineal trauma in all births has been maintained at a rate below 1%.
